# Preliminary Observations

**Published:** 1830-10-01

**Authors:** 


### XXXVII.

### ST. JOHN LONG.—CORONER'S INQUEST.

Lethiferque per orbem dicor.

THE race of the above charlatan is run! He and his suborned, bribed auxiliaries of the venal press, may now lay down their heads on their pillows, and sleep if they can, after the perfect conviction that they have sent prematurely to their long homes, some of the most amiable beings of the human race! We were the first to expose the quackeries of St. John Long, and our readers are well aware of the vituperation which that exposure drew on us from venal journals in the pay of the tamperer with human life. The day of retribution has come round—and the remorse which the satellites of corruption must feel, need not be heightened by any rhetoric from us. We have spoken of REMORSE; but we much doubt whether these gentry are susceptible of any thing of that kind, beyond their own pockets. Their pecuniary interest is now pretty nearly at an end;—for whether any trial for manslaughter or damages may ensue, is immaterial;—the ignorant and reckless deceiver must hide his head and quit the kingdom for ever.

We place the record of this Coroner's inquest on our pages, for several reasons;—but principally for the purpose of enabling our medical brethren to exhibit it to such of their patients as are desirous of putting themselves in the hands of charlatans!—We shall, however, divest it of all unnecessary evidence and irrelevant matter.

### Coroner's Inquest.

On Saturday morning, at 11 o'clock, an inquisition was commenced before Mr. Stirling, coroner, and a highly respectable jury, at the Gate-house, in the Hampstead-road, on view of the body of Miss Catherine Cashin, who, it was alleged, had died in consequence of the treatment which she received at the hands of Mr. St. John Long, a person residing in Harley-street, Cavendish-sq. professing to be a medical practitioner. The investigation excited the deepest interest in the neighbourhood; and the feeling of anxiety that existed was by no means lessened by the circumstance of the sister of the above-named young lady, who was also a patient of the same person's, having died on Saturday morning, a few hours only before the coroner and jury assembled. A solicitor took a seat at the coroner's table, and stated, in answer to a question from the Court, that he attended for a party very deeply interested in the inquiry.

Mrs. Mary Anne Roddis, a lady-like and extremely intelligent woman, was called in and sworn, and gave the following statement, which was interrupted at intervals by the agitation of the witness:—I reside at No. 32, Mornington-place, Hampstead-road, and am a married woman. My husband is a wine-merchant. I knew the deceased about two months; she and her mother and sister lodged in my house. When I first knew the deceased she was in perfect health, and she continued so until about 10 or 11 days ago. Yesterday (Friday) week I was requested, by the deceased's mother, to accompany her daughter to Mr. Long's in Harley-street, and to state the fears she had respecting the wound in the deceased's back. I complied with Mrs. Cashin's request, and accompanied the deceased, and saw Mr. Long, and drew his attention to the state of her back. He looked at it, and said it was precisely as he wished it to be. The next thing I can state is, that on Saturday morning I was called up to the apartment of the deceased, and found her in the greatest agony. I then looked at the back. Need I state, Sir, the appearances of the back, and the opinion I formed?

The Coroner.—You may state any fact, but I will not ask you to give any opinion.

Witness.—It is impossible for me to describe here the state of her back minutely. It was in a very violent state of inflammation, and she was troubled with unceasing sickness. In the afternoon of the same day I wrote, in Mrs. Cashin's name, to Mr. Long, requesting his immediate attendance. He came between five and six o'clock in the evening. He then looked at Miss Cashin's back, and said he thought it was in a very good state, and that he would give 100 guineas if he could produce a similar wound upon the persons of some of his patients. I pointed out one particular spot upon her back, and asked him what could cause its appearance; and he said it was in consequence of inhaling, which was a part of his system, and unless those appearances were produced, he could expect no beneficial result. He then inquired what I had done, and I told him I had applied a poultice, and given a saline draught to allay the irritation of her stomach. He said I could not have done better, and that he should make no alteration. I then begged him to order something that would allay the irritation of the stomach and bowels. He said it was of no consequence, as that irritation was the effect produced by his system, and it would be of ultimate benefit to her. I then begged that he would at least give her a composing draught, to which he replied that a tumbler of mulled port wine was a better composing draught than all the doctors in the world could make, for that he hated the very name of physic. I objected to this, but he insisted on its being given. I gave her a wine glass full, which was immediately rejected by the stomach. By Mr. Long's permission I continued the poultices, but she gradually got worse. On Sunday morning last he came again, and said that doubtless I knew better the state of the wound than he did, because I constantly applied the poultices. On removing the poultice on this occasion, I again pointed to the dark spot in the wound, and he said that probably there would be a number of boils come out, and that it was what he wished to produce; and that she was going on as well as he could possibly wish. I then named the exhausted state of the patient, and again pressed his attention to the sickness, as well as did the deceased's mother. He said he had a remedy with him, but that he would not apply it then, because he rather liked the sickness than otherwise. He then ordered her to have a little rhubard and magnesia, which was immediately given. Between 11 and 12 o'clock on Sunday night he came again, and gave her some medicine which he had brought with him, but which was immediately thrown off the stomach. On my saying that I thought her in a dangerous state, he said my fears were perfectly groundless, and because of my ignorance of his system it was that I was alarmed; and that she would be perfectly well in a few days. She passed a very restless night, and on the following morning I was still more alarmed at the increased inflammation of the wound. The deceased's mother and brother-in-law expressed a great desire to have further advice, and Mr. Brodie was sent for, who saw her about six o'clock in the evening, and ordered a poultice to be applied immediately, and some saline draughts. The sickness was stayed by Mr. Brodie's prescription. Her night was very bad. On the following morning at 7 o'clock, I gave her a saline draught, and in half an hour afterwards she took a small cup of coffee with dry toast. I left her with the nurse, under the impression that she was drowsy. During the time I was at breakfast, a bell rang violently, and I immediately ran up stairs and saw the deceased in the agony of death. Mr. Foulkes, a neighbouring surgeon, was sent for immediately. I tried to get a tea spoonful of brandy into her mouth, but her jaws were quite set, and she immediately breathed her last. [Here the witness was deeply affected, and it was with difficulty that her husband, who sat by her side, could with his utmost attention keep her from fainting.] I did not state in its proper place, that after Mr. Brodie had seen her on Monday evening, Mr. Long called. On hearing that Mr. Brodie had been called in, he said it was perfectly unnecessary, for that no person could be doing better than Miss Cashin was. Mr. Long did not come or send after that.

The solicitor (Mr. Adolphus, for Mr. Long) said he wished to ask the witness whether the diet which Miss Cashin took was in accordance with the directions of Mr. Long on that head.

Witness.—Mr. Long said he wished his patients to eat and drink whatever they liked; so that there was no restriction.

The Coroner.—What did she eat or drink?

Witness.—She had no appetite for any thing. We tried a variety of things which we thought likely to stay upon her stomach, all of which I told Mr. Long of, and he approved.

Solicitor.—What sort of appearance did the spot of which you have spoken bear?

Witness.—It had a very dark and angry look.

A Juror.—Did both the sisters reside in your house, and did Mr. Long visit them both?

Witness.—Yes; latterly, when Miss Cashin became so ill, he was sent for; but before that they visited Mr. Long. He does not usually visit his patients—they go to him. Miss Cashin used to go with her sister before she became a patient.

Juror.—Is there not some kind of secrecy observed in Mr. Long's practice,—I mean, are not his patients bound to secrecy in some way?

Witness.—I understand there is something of that sort. I do not know whether Mr. Long was made acquainted with the nature of Mr. Brodie's prescription when he came on Monday evening. It was sent to Mr. Foulkes's to be prepared. I do not know at what particular time Miss Cashin first became a patient of Mr. Long's. She had nothing the matter with her; she had no complaint; but she said Mr. Long's treatment was to prevent any disease in future.

Solicitor.—Was she aware that her sister's back was bad?

Witness.—I do not know. Mr. Brodie saw the deceased's back.

Solicitor.—What opinion did you form of it when you saw it?

Witness.—My impression was, that there was very violent and extensive inflammation, which if not stopped would produce very serious consequences, and I told Mr. Long so; and his reply still was, that it was part of his system.

Solicitor.—Did you point out the spot you have spoken of to Mr. Brodie?

Witness.—I did not call his attention to any particular part of the wound.

Solicitor. Why did you not?

Witness.—Because I thought it would be presumptuous in me to do so to so eminent a man as Mr. Brodie.

Solicitor. But why should you have mentioned it to Mr. Long, and not to Mr. Brodie?

Witness.—If I must answer that question, it was because I thought Mr. Long a very ignorant man, but I knew Mr. Brodie to be otherwise.

By the Coroner.—The inflammation extended all over the back on both sides of the vertebræ. It was at the top part, between the shoulders. The ladies were strangers to me before they came to lodge in my house.

Juror.—When was the wound made?

The Coroner.—The witness cannot know, because she did not see it made.

The witness, whose examination lasted nearly two hours, then signed the deposition and withdrew.

Mr. Benjamin Collins Brodie sworn.—I reside at No. 16, Saville-row, and am a surgeon. I never saw the deceased but once during her life-time. I was sent for on Monday last to 32, Mornington-place, and I went between five and six o'clock. I first saw in the drawing-room a young lady who appeared to be labouring under an affection of the chest. I went up to a bed-room, and there I saw a young lady who was said to have a wound in her back, which it was wished I should see. I looked at her back and discovered a slough, which might be as large as the palm of my hand, all round which the skin was discoloured to a considerable extent, as if it had been inflamed, and was now threatening to become a slough also. Her stomach was very much disturbed, and I was told that she brought up whatever she swallowed directly. I prescribed what in my judgment were suitable remedies, and said I would call on the following day, believing at that time that, although she was very ill, she was not in immediate danger. I was told that the inflammation in the back which produced the sloughing (which term, perhaps, I should explain to the jury, is synonimous with mortification, or nearly so) had been produced by an application made by a Mr. Long, who had been consulted, but of this I have no actual knowledge myself. On the following day (Tuesday) I called again at the house, and found that the young lady had died that morning. I requested that I might see the body, and on examining the back, I found that the sloughing had very considerably extended, and I concluded had been the cause of her death. That is all I know and all I can say. I have no knowledge of how the wound was produced.

Solicitor.—Might not such a wound have been produced by a blister?

Witness.—On a person of very susceptible constitution it might. I was told that she was not ill at all before the wound was made, but that it was made to prevent her from going into a consumption like her sister. On that subject I was informed her mother was very anxious.

By the Solicitor.—Any considerable stimulant applied to the skin of a person of extremely susceptible constitution may possibly produce mortification. A common blister might produce it, but it is not likely. I did not see what application had been made to the wound, but was informed that it had been poulticed. I was informed that her stomach would not bear medicine, but what I gave her stayed. The wound in her back was quite sufficient to account for the state of her stomach. The slough, as I saw it on the following day, was quite sufficient to destroy any one.

Solicitor.—Did you observe any particular spot?

Witness.—There was a red surface the size of a good large plate, but the sloughing was not so extensive. The centre of the wound was quite black, the rest red.

Solicitor.—If you had seen her on Friday, do you think it likely you could have done her any good?

Witness.—I think it very likely, though to be sure I am unacquainted with her constitution, and therefore cannot speak with certainty.

A Juror.—Do you think the application made by Mr. Long to her back was the cause of her death?

Witness.—I think so, from what I have heard.

Juror.—Would inhaling produce such a wound on the back?

Witness.—Not alone.

Juror.—Is there not some secret in Mr. Long's practice? I mean, does he not profess great secrecy, and bind down his patients not to divulge his mode of treatment?

Witness.—I have heard so.

Solicitor.—Might not eating a large quantity of plums cause inflammation in the bowels and stomach, and might not that inflammation extend to the back, and show itself eventually in the form in which you saw it? Is it not possible?

Witness.—I don't know what is possible, but I never saw any thing of the kind. It could not produce such a slough as that upon her back.

A Juror.—You did not, I think, consider her in danger when you first saw her?

Witness.—I thought her very ill, but not in immediate danger.

Juror.—Could the appearances you have described have been produced by inflammation of the bowels?

Witness.—Certainly not. The sloughing could not have been produced by that.

By the Solicitor. The sickness and disturbed state of the stomach would not have occasioned the slough, but the slough was quite sufficient to account for the sickness. The effect of any external application would depend, of course, in a great measure, upon the constitution of the patient. I was told the deceased was in good health ten days ago.

A Juror.—Do you think an application of the kind that has been mentioned likely to prevent consumption?

Witness.—Certainly not. I cannot imagine how such a thing could be.

The Coroner.—Would you consider it warrantable practice to produce such a back in order to prevent consumption?

Witness.—I should not.

A Juror.—Would you think a tumbler of port wine the best composing medicine for her?

Witness.—It might have been very good for her, if the stomach would have borne it; but it certainly was not calculated to quiet her stomach as it then was.

Juror.—Did you not say she had been murdered?

Witness.—I did not mean murdered in the literal sense; but I believe I did say something of that kind; and my opinion was, that she had, in consequence of the treatment she received.

Solicitor.—You have said that the effect produced by any external application would greatly depend upon the constitution of the patient. Then Mr. Long's system, although not beneficial in this instance, might be so in others?

Mr. Brodie (smiling and shaking his head.)—I cannot say any thing about that.

A Juror.—If you had visited Miss Cashin before, and had seen the sloughing increase, would you not have thought there was danger?

Witness.—Certainly.

Juror.—Would you have thought she was going on well?

Witness.—No. I have not seen the body since the day she died, but I have no doubt that the sloughing was the cause of her death.

The coroner and jury, with Mr. Brodie and Mr. Wakley, then went to view the body, and on their return several of the jury expressed a strong desire to have the body opened; one of the gentlemen observing, that although there could be very little doubt as to the cause of her death, and although they would willingly spare the afflicted mother any further trial of her feelings, yet the case was one of such vast public importance, every possible information must be obtained.

An elderly person sitting at the table, who said he was a surgeon, said he hoped the jury would hear evidence that Mr. Long had to adduce on his own behalf. He (the speaker) could bring forward testimony in contradiction of what had been stated, and he himself knew of 50 cases within these two months in which Mr. Long's system had been tried, and not one instance of a failure.

The solicitor said, Mr. Long's professional reputation was at stake, and he trusted the jury would hear both sides.

Mr. Sweetman, brother-in-law of the deceased, said there would be no objection on the part of the relatives to having the body opened, as it was necessary for the furtherance of justice.

It was then directed that the body should be opened immediately, and the inquest was then adjourned until Monday (this day.)

Mr. Long was in the house during the inquiry, and was accompanied by Mr. Van Butchell, and one or two other persons of advertising celebrity.

A great part of the second day of the inquest was spent in very unnecessary altercations—and the evidence of Dr. Thomson was any thing but calculated to enlighten the minds of a non-professional jury. We shall select those parts of this day's proceedings which were at all ad rem.

Mr. Adolphus.—I believe, Sir Francis Burdett, you have had some means of making yourself acquainted with some portion of Mr. Long's mode of practice?

Sir F. Burdett.—I have been admitted to Mr. Long's house, and have seen him practise several times.

Mr. Adolphus.—Did his practice appear to you to be dangerous, or likely to be beneficial to his patients?

Sir F. Burdett.—I should think there was not the slightest degree of danger in it certainly.

Mr. Adolphus.—Were the manners of Mr. Long those of a mild and humane man, or those of a cruel and unfeeling practitioner?

Sir F. Burdett.—Quite the contrary of the latter certainly. But perhaps I had better state what passed. I went to Mr. Long in consequence of hearing that he had cured two persons of *tic douloureux*, with a view to see whether any relief could be afforded to the Marquis of Anglesey, and, from what I saw, was so convinced there was no danger in his mode of treatment, that having the gout in my hand, I desired Mr. Long to try what effect it might have upon me, more, however, for the purpose of having an opportunity of reporting to Lord Anglesey that there was no danger in the operation, than the hope that it would do any good for the gout. I did report to Lord Anglesey the result of my observation, and I believe he would have had recourse to Mr. Long, if he had not got better just at that time. So satisfied was I from what I saw, and from what I heard from persons attending Mr. Long for advice, of the beneficial effects of his practice, that one or two other individuals put themselves under his care at my recommendation.

Mr. Wakley.—Pray, Sir Francis Burdett, what operation was the Marquis of Anglesey to undergo?

Sir F. Burdett.—I only know that he was to have an outward application by friction. I know nothing of the ingredients of which the preparation that was to be used was composed. I know he operated on others who were labouring under the same complaint, and they told me they had received benefit.

Mr. Wakley.—Do you know, of your own knowledge, of any one who has been cured by Mr. Long?

Sir F. Burdett.—No, I do not. Lord Sligo told me he had been cured of the gout by Mr. Long, and I dare say he was for the time, but how long it would last I don't know.

Mr. Wakley.—Did you receive any benefit from the application?

Sir F. Burdett.—None whatever.

Mr. Wakley.—When did you first see Mr. Long?

Sir F. Burdett.—About two months ago, I think; but I am not sure.

Mr. Wakley.—Was there any injunction imposed upon you as to secrecy?

Sir F. Burdett.—None, that I recollect.

Mr. Wakley.—Did you not sign a book?

Sir F. Burdett.—I believe I signed something, but I am sure I do not now recollect what it was. I believe, however, there was something said about keeping it secret.

Mr. Wakley.—Can you judge of the ingredients of the preparation from what you saw of its application?

Sir F. Burdett.—No, I cannot. I do not know what effect it produced, except that it made the skin look red and angry, as is generally the case where friction is used. I saw no appearance of mortification. I saw the process of inhaling performed, but I do not know the qualities of the gas inhaled.

Mr. Wakley.—Have you ever made medicine your study?

Sir F. Burdett.—Oh dear, no.

Mr. Wakley.—Should you be able to distinguish a glass of water from a glass of prussic acid by the appearance?

Sir F. Burdett.—I don't think I should.

Dr. Alexander Thomson was next sworn.—He stated that he lived at No. 70, George-street, Euston-square. He had examined the body of the person reported to have been called Miss Catherine Cashin. He had examined the external parts of the body wholly, but his examination of the internal parts had been confined to the abdomen and thorax. He wished and was about to open the head, when he was informed that it was objected to by the family. He was informed that the mother was lying up stairs in a dreadful state of mental affliction, and that she gave a decided negative to the proposal to proceed with the examination of the head and spine, and of course he was obliged to desist. Mr. Wildgoose, a surgeon, who appeared to conduct the examination of the body on behalf of Mr. Long, and witness, had taken notes of the result of their examination, and they had jointly drawn up a report, which, with the permission of the jury, he would read for their information.

Juror.—Was there not what is called a slough or sloughing in the back?

Dr. Thomson.—I am sorry you have put your question in that way, because it places me in a very delicate situation. *There was no sloughing. By sloughing we mean the coming away of a dead part.*

Juror.—Was there nothing of that kind in the back?

Dr. Thomson.—I should say, certainly not. The appearances in the abdomen and thorax were the same precisely as I discovered in examining the body of a young lady who died some time ago, at Chelsea, from taking colchicum in too large a quantity. It is by no means an uncommon practice now with the junior branches of the profession to produce what is called counter-irritation, by applying stimulants to one part in order to relieve another. This is found beneficial in certain systems. I again repeat, however, that from what I have seen of the young lady, I cannot found any decided opinion without examining the brain and spine. I have seen death occasioned with less inflammation and general disorganization of the stomach than appeared in this instance, always excepting the appearances in the back, for on no occasion have I seen a back in such a state. The inflammation, therefore, I say, in the stomach was sufficient to have caused death, and if I had found no other appearances than those in the back, I should say that the state of that part was also sufficient of itself to have produced death.

Mr. Adolphus. — Taking all appearances and all circumstances into consideration, would you say that death was occasioned by the wound in the back?

Dr. Thomson.—Taking my means of information from the examination I was enabled to make, I should say no; but I should, I once more repeat, have been able to state a decided opinion if I had been allowed to examine the spine and brain.

A Juror.—If you had seen the deceased 10 days ago perfectly well, would you have caused, by any application, such a state of the back?

Dr. Thomson.—No, unless I wished to kill my patient. Such a thing was never done by any medical man that I know of.

Juror.—Supposing the young lady to have a sister who was consumptive, and that she herself, although in good health, was afraid of being similarly afflicted, and applied to you, would you have produced such a sore?

Dr. Thomson.—Certainly not. I would never introduce such a sore for the purpose of producing counter-irritation.

Mr. Wakley.—If you had proceeded to the examination of the brain and spine and found no disease there, what would you then have thought the cause of death?

Dr. Thomson. — The disease in the back.

The Coroner said, the only way would be to have the spine and brain examined now, if the Jury thought fit.

Dr. Thomson, in continuation, said, he never saw such a sore as that in the deceased's back, although he had known red hot irons to be applied to the skin to produce counter-irritation. He wished very much to pursue the examination of the body.

The witness here produced a strip of the skin which he had taken from the deceased's back, and which was handed round to the jury, and inspected by the numerous medical men present, Dr. Thomson observing, that any medical gentleman would perceive that there was no slough in it, nor was there any disorganization.

Upon the above evidence we are compelled to make a remark or two. Under what species of mental confusion, or rather delusion, Dr. Thomson laboured, when he denied that the parts were disorganized or in a state of slough, we cannot imagine. We saw the parts. They *were* disorganized. *They were in a state of slough.* Not a single medical tyro in the profession could have pronounced them to be in any other state. Dr. Thomson indeed evinced some lack of discretion, or at least of *tact*, in his evidence. A medical audience could scarcely have understood his technical refinements, much less a jury of non-professional people. Mr. Brodie's description of the parts was perfectly correct—that of Dr. Thomson was erroneous.

The next day, Tuesday, the body was disinterred, and examined in the vaults of Moorfields Chapel. We were present at this examination. The brain and its coverings were perfectly natural. The coverings of the spinal marrow were red, but, on the most accurate investigation, with glasses and the naked eye, it was evident that the red colour was a tinge produced by the blood, and not the product of inflammation. No vessels were perceptible.

The third day's inquest revealed the results of the disinterment and re-dissection in the vaults of the Catholic Chapel in Moorfields. A considerable degree of decomposition had taken place, and the smell of the body and of the vaults generally was most offensive and oppressive. It is useless to multiply the statements of the medical witnesses. They were all in accordance, with the exception of Mr. Wildgoose, whose depositions shew pretty evidently that he was at the dissection as the friend of Mr. Long.

Dr. John Hogg sworn.—Was present yesterday, in the vault in Moorfields chapel, and assisted in examining the head and spine of the deceased. The first thing that struck him as remarkable was the state of the back. It presented between the shoulder-blades a very large kind of eschar; it appeared as if it had been scorched by fire. The body itself was not at all emaciated, but muscular, symmetrical, and in many respects well formed. Dr. Thomson, witness, and others, proceeded to examine the spine; obtained a full view of the spinal cord. There was certainly an appearance on the sheath of the spinal cord, opposite to where the external sore was, of discolouration. This was very minutely examined. On removing it to the day-light, it exhibited a crimson appearance. The other part of the sheath was of a more natural colour. On opening the sheath, it was evidently thickened at the part where it was discoloured; but had no appearance of disease. The cord itself was not at all affected. The examination was next directed to the head. On removing the skull-cap, the brain presented an unusually firm and healthy appearance; portions of it were removed and examined as to its structure, which exhibited a state of perfect health. The brain was perfectly sound at the basis, and at the origin of all the cerebral nerves, and all the nerves emanating from the brain itself. Not having been present at the former examination, it was difficult for him (witness) to form an opinion as to the cause of the death, but should say that the violence which had been done to the nervous system was quite sufficient to cause death, particularly in the case of a nervous and delicate young lady.

By a Juror.—The sheath of the spinal cord was discoloured from external causes. The opinions of the different gentlemen present at the examination were not communicated to each other; each gentleman made and brought away his own observations. As a professional man he (witness) should certainly not create such a wound on a healthy person. Had not the slightest idea what such a wound could have been made for. Should say decidedly that the lady had not been labouring under the effect of any disease except that of the wound produced on the back, as far as he saw: in death even the body had the appearance of health. Had never seen a more beautifully formed body. Had never seen such a wound produced on an unhealthy person, and should be sorry to produce such a one. If the mother of a patient had gone to him, and said other children of her's had died of consumption, and that she was afraid of the one produced falling a victim to the same disease, there were prophylactics that might be had recourse to, but he should never think of making such a wound.

Dr. James Johnson examined.—I reside in Suffolk-place, Pall-mall east. I was present at the examination of the body yesterday. The sheath of the spine was discoloured, but the whole was not thickened. I do not think the redness was the effect of inflammation. I think it was merely tinged by the blood itself. I conceive that the patient died from several causes, the primary one being the local inflammation, which produced incipient gangrene. The next effect was fever resulting from that inflammation; and, thirdly, the inflammation of the membranes of the stomach and pleura connected with the fever. I suppose the fever to have been produced by the local inflammation, and to have been symptomatic of that inflammation and of the incipient gangrene. I think death was produced by these circumstances combined, all arising from the inflammation in the back. The cause of this inflammation is kept a secret, therefore we can only guess at it.

A Juror.—Could you by analyzation discover what the preparation was which was used?

Dr. Johnson.—No; we might perhaps discover some portions of the preparation, but we could not ascertain what the composition was. I did not examine the body internally. I should not have made such a wound in any disease, and certainly not in a healthy subject.

The following morçeau of evidence will explain the observation we made above.

Mr. W. Wildegoose, surgeon, sworn.—Having read the depositions that had been taken, found it difficult for him to say what the cause of death had been. Having nothing else to go by than the appearances after death, he was bound to suppose that the injury which had been inflicted had been the cause of the death, but he could not swear that it was. The internal surface of the stomach and duodenum were inflamed. Inflammation of the stomach could not exist for any length of time without killing the patient, independent of any other injury or disease. Had seen the back, and if he (witness) had nothing to go by but the back alone, he should not have supposed it would have caused death.

By a Juror.—Could not tell whether the inflammation of the bowels could have been occasioned by the state of the back or not: it might or might not by sympathy. Had been in the habit of examining bodies after death. The appearance of the back was somewhat like as if lunar caustic had been applied to the part. The skin was mortified, but the muscles were uninjured. Had seen such injuries before. Had seen deeper ulcerations produced by caustic. Had never seen an injury intentionally produced to such an extent on the back of any individual. Had he been called in, and supposing the inflammation of the stomach to have been sympathetic, he might have done something for the relief of the patient. Had no means of knowing what had caused the wound. He should not like to see such a wound on the back of any patient of his. Knew nothing of Mr. Long's mode of treatment.

By Mr. Adolphus.—Knew Mr. Long. Had heard from very respectable people that he was a successful practitioner. Had known him for some years, and seen many patients go to him.

By a Juror.—Did not know until this investigation had begun, whether the persons he had seen go to Mr. Long were patients or visiters, as he had never been inquisitive respecting Mr. Long's affairs; believed part of his plan of treatment to be that of counter-irritation; that was, to produce an external illness for the purpose of drawing off an internal disease. Would not have made a wound so large as this on the back under the circumstances. From what he had seen since death, he did not think he should have been justified in making so large a wound. Consumption consisted of tubercles of the lungs. His conduct in treatment would be much influenced by a mother, or an old woman, saying other branches of a patient's family had died of a consumption, but not so much as to be induced to make such a large eschar as this was.

By Mr. Wakley.—Mr. Long was not studying the profession, nor was he an authorized surgeon when he (witness) first knew him. Could not say how many years, but it was before he began this system. (Great laughter.) Did not know that he had received a medical education. Did not know what trade or profession he was of some few years ago. A short time ago he was a painter. He was not now of the medical profession, but was what was called a professed curer of consumption.

The inquest was adjourned till Friday, the 27th August. We saw some of the boobies who pinned their faith on St. John Long, waiting in the anti-rooms—among the rest, a superannuated medical numbscull, who came hobbling to give his testimony to the wonder-worker of Harley-street!

The lateness of the period at which this interesting and melancholy inquest took place, disables us from doing more than placing the principal facts on record. In another article, we shall take an opportunity of offering some strictures on the evidence, medical and non-professional, which came out on this memorable occasion, and we hope that the said strictures will embrace some important and interesting topics. Without presuming to determine the final results of this inquest, we venture to predict that it will lead to some of a very salutary nature. We think it will rouse the legislature to a Parliamentary enactment that may check the career of charlatanism, by pointing attention to a quack whose unblushing pretensions have exceeded any thing yet on record! It is astonishing to find that this ignorant pretender is peculiarly fostered and patronized by the ARISTOCRACY of this country, and by people whose education and rank might indicate some portion of reason and common sense. But more of this anon.

